# Landscape metrics as predictors of hydrologic connectivity between Coastal Plain forested wetlands and streams

**DOI:** 10.1002/hyp.11433

**Published:** 2018-02-20

**Authors:** Steven M. Epting, Jacob D. Hosen, Laurie C. Alexander, Megan W. Lang, Alec W. Armstrong, Margaret A. Palmer

**Affiliations:** ^1^ Chesapeake Biological Laboratory University of Maryland Center for Environmental Science Solomons MD 20688 USA; ^2^ National Socio‐Environmental Synthesis Center University of Maryland 1 Park Place, Suite 300 Annapolis MD 21401 USA; ^3^ Environmental Protection Agency, NCEA Washington, D.C. 20460 USA; ^4^ United States Department of Agriculture Forest Service Beltsville MD 20705 USA

**Keywords:** catchment, geographically isolated wetlands, hydrologic connectivity, nonperennial

## Abstract

Geographically isolated wetlands, those entirely surrounded by uplands, provide numerous landscape‐scale ecological functions, many of which are dependent on the degree to which they are hydrologically connected to nearby waters. There is a growing need for field‐validated, landscape‐scale approaches for classifying wetlands on the basis of their expected degree of hydrologic connectivity with stream networks. This study quantified seasonal variability in surface hydrologic connectivity (SHC) patterns between forested Delmarva bay wetland complexes and perennial/intermittent streams at 23 sites over a full‐water year (2014–2015). Field data were used to develop metrics to predict SHC using hypothesized landscape drivers of connectivity duration and timing. Connection duration was most strongly related to the number and area of wetlands within wetland complexes as well as the channel width of the temporary stream connecting the wetland complex to a perennial/intermittent stream. Timing of SHC onset was related to the topographic wetness index and drainage density within the catchment. Stepwise regression modelling found that landscape metrics could be used to predict SHC duration as a function of wetland complex catchment area, wetland area, wetland number, and soil available water storage (adj‐R ^2^ = 0.74, p < .0001). Results may be applicable to assessments of forested depressional wetlands elsewhere in the U.S. Mid‐Atlantic and Southeastern Coastal Plain, where climate, landscapes, and hydrological inputs and losses are expected to be similar to the study area.

## 1 INTRODUCTION

Connectivity has long been recognized as an important concept in landscape ecological research and planning (Merriam, 1984; Taylor, Fahrig, Henein, & Merriam, 1993; Turner, 1989). As paths of movement for organisms, connectivity between habitats has played a prominent role in the study of metapopulation dynamics, gene flow, extinction risk, and reserve design (Fagan, 2002; Fahrig & Merriam, 1985). Hydrologic connectivity, or the water‐mediated transport of matter, energy, and organisms within or between elements of the hydrologic cycle (Pringle, 2001), has also been a major focus due to the fact that water is a universal driver of landscape structure (Larsen, Choi, Nungesser, & Harvey, 2012; Ward, Tockner, Arscott, & Claret, 2002), ecological function (Pringle, 2003) and ecosystem services (Brauman, Daily, Duarte, & Mooney, 2007). Hydrologic connectivity can be highly dynamic, particularly between transitional landscape elements such as wetlands, which vary in size and degree of hydrologic connectivity depending on their landscape position as well as on net inflows and outflows from ground, surface, and atmospheric water (Bracken & Croke, 2007).

Attributed in large part to their dynamic nature, wetlands perform a number of important hydrologic, biogeochemical, and habitat/food web functions with local and regional effects (McLaughlin & Cohen, 2014; Sharitz, 2003). Wetlands supply materials such as water and organic matter (source function), remove harmful materials such as excess nutrients and pathogens (sink function), and provide habitat or refugia for organisms such as fish and aquatic insects (Leibowitz, Wigington, Rains, & Downing, 2008; Rains et al., 2016). Among depressional wetlands, longer wetland hydroperiods and occasional surface water connections to permanent waters have been linked to higher species richness (Snodgrass, Bryan, Lide, & Smith, 1996) and higher net primary productivity (Cook & Hauer, 2007). Wetland area within a watershed has been shown to be significantly related to flood control (Mitsch & Gosselink, 2000) and reduced nitrate concentrations in groundwater and surface water (Phillips, Denver, Shedlock, & Hamilton, 1993). The type, magnitude, and scale of these functions depend considerably on the degree and mechanism of hydrologic connectivity between wetlands and other landscape elements (Cohen et al., 2016; Leibowitz, Mushet, & Newton, 2016; Marton et al., 2015).

Processes at the landscape scale such as watershed runoff response downstream of depressional wetlands are also affected by hydrologic connectivity. Runoff is influenced by the spatial relationship between runoff‐generating areas, flow pathways, surface water storage, and the catchment outlet (Ali et al., 2015; Nippgen, McGlynn, Marshall, & Emanuel, 2011; Shaw, Pietroniro, & Martz, 2013). Thus, changes in land use that alter hydrologic connectivity (e.g., urbanization, agriculture, mining, and channelization) can result in dramatic changes in runoff and the transport of pollutants to downstream waters (Marton et al., 2015) due to loss of wetland water storage and filtration functions (Zedler, 2000). Agricultural drainage through ditching and tiling has led to the greatest loss of wetlands globally (Bartzen, Dufour, Clark, & Caswell, 2010; Blann, Anderson, Sands, & Vondracek, 2009) and altered hydrologic connectivity over sufficiently large scales as to extirpate some wetland species (Jenkins, Grissom, & Miller, 2003). Despite significant loss of wetlands and dramatic changes in hydrologic connectivity, recent use of high resolution imagery has shown that remaining wetlands and small streams in ditched agricultural landscapes may be far more numerous than previously thought (Lang, McDonough, McCarty, Oesterling, & Wilen, 2012), and the plugging of ditches to restore wetlands can result in even greater surface hydrologic connectivity (SHC; Jones et al., 2017; McDonough, Lang, Hosen, & Palmer, 2015). Interest in preserving and restoring wetlands as well as protecting downstream waters has spurred interest in understanding the dynamics of SHC. Researchers are asking when and where temporary flow paths between features of the landscape exist and how important these dynamic connections are in ecological, chemical, and hydrological contexts.

**Figure 1:**
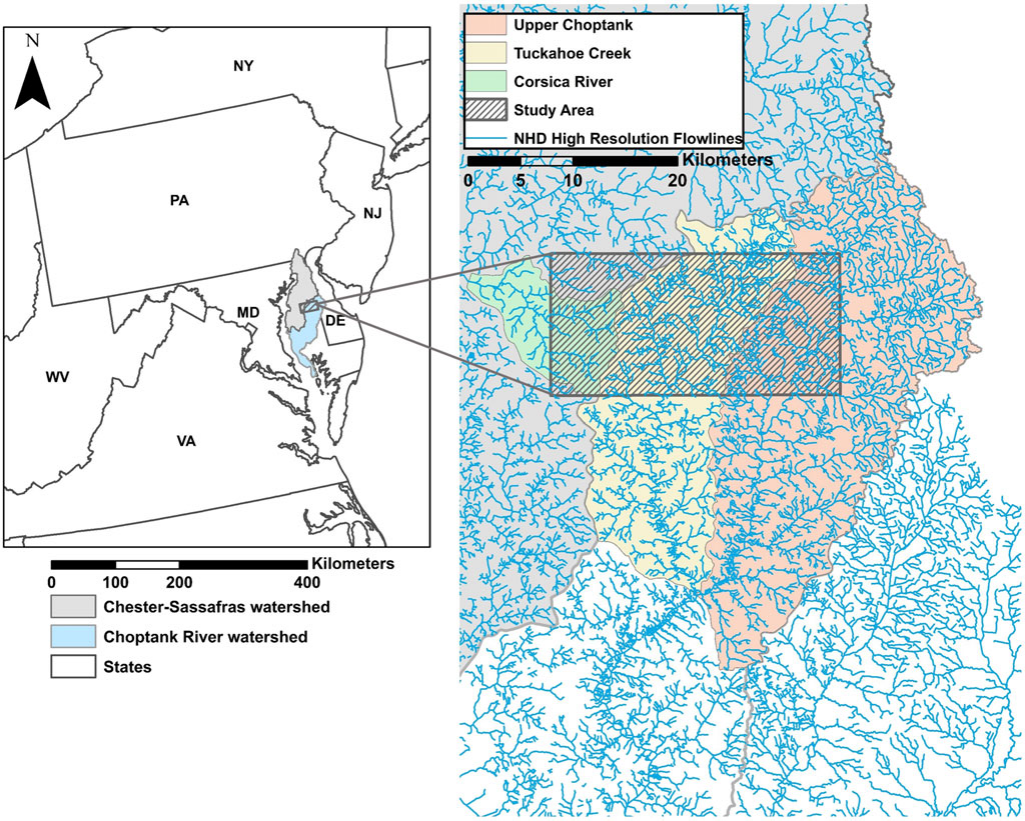
Location of the study area within the upper portions (Upper Choptank, Tuckahoe Creek) of the Choptank River watershed and Corsica River watershed

**Figure 2:**
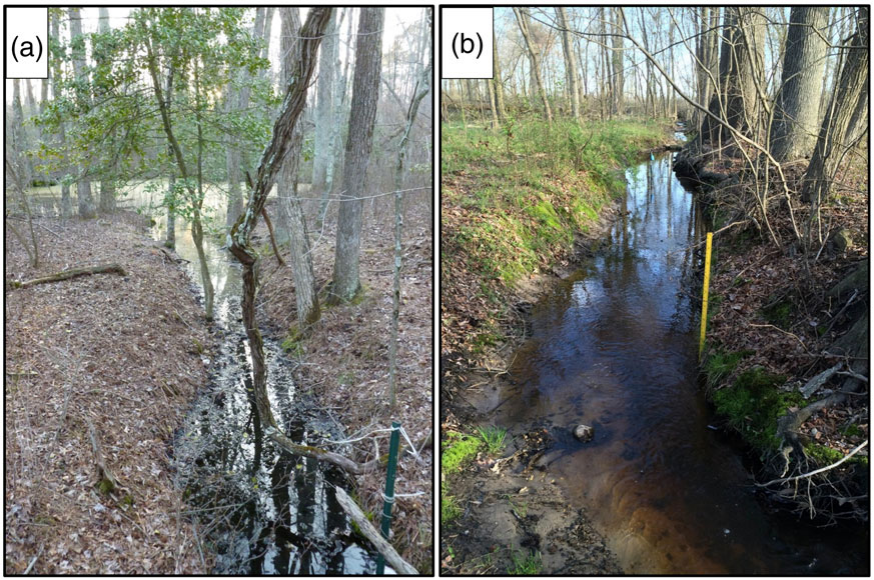
Nonperennial streams connect many forested wetlands to downstream perennial waters via surface flow. Dates pictured: 12 April 2014 (a), 19 April 2014 (b)

Here, we focus on the seasonal hydrodynamics of Delmarva bays in the eastern Delmarva Peninsula (Figure 1) with the goal of advancing our understanding of the relationship between landscape characteristics and the hydrologic connectivity between these wetlands and the surrounding stream network. Delmarva bays are geomorphological features characterized by their depressional shape. They have previously been classified as “geographically isolated” wetlands (Tiner, 2003)—wetlands completely surrounded by uplands—as the majority lack clearly defined surface water inlets or outlets. Despite this classification, the surface water extent within individual bays varies seasonally and inter‐annually with rainfall and antecedent conditions (Pyzoha, Callahan, Sun, Trettin, & Miwa, 2008). During wet periods, surface water levels frequently exceed storage capacity and individual basins frequently merge to form large wetland complexes (Sharitz & Gibbons, 1982) that outflow to stream networks (McDonough et al., 2015). Additionally, the Delmarva Peninsula is characterized by hand‐dug ditches created in the early to mid‐1900s to drain wetlands for agriculture and mosquito control (Figure 2). Many of these ditches now function as temporary streams (i.e., streams in which surface flow is absent for some portion of the year) connecting wetlands to the perennial stream network. Using a semi‐automated stream mapping approach on the basis of light detection and ranging (LiDAR) digital elevation maps, Lang et al. (2012) reported that 53% of semi‐natural palustrine wetlands in a Maryland Coastal Plain watershed were directly connected to streams and 60% were stream‐connected within a 10‐m stream buffer.

Although distance‐based methods may provide a reasonable first‐order approximation of structural physical wetland‐stream connectivity at regional or national scales, more accurate approaches are needed to predict functional SHC at catchment scales. Given that surface water connections are easier to assess than groundwater dynamics, the importance of hydrologic connections from wetlands to downstream waters is often based on estimates of SHC. Paired with a mechanistic understanding of the drivers of local hydrology, recent advancements in remote sensing and geographic information system (GIS)‐based methods provide an opportunity to more accurately map the spatial extent of streams and wetlands and predict the degree of connectivity between water features across the landscape. These advancements could also provide an important tool for managers and regulators in need of estimates of hydrologic connectivity. The goal of this study was to develop an approach for predicting connectivity using field and GIS‐derived landscape predictor metrics representing drivers of connectivity duration and timing.

The specific objectives of this study were to (a) quantify seasonal variability in SHC patterns between Delmarva bay wetlands and perennial streams from field observations; (b) develop predictive metrics from hypothesized landscape drivers of wetland‐stream SHC; and (c) model cumulative SHC duration, seasonal connection onset dates, and seasonal connection offset dates as a function of landscape predictor metrics.

## 2 STUDY SITES

### 2.1 Location and characteristics

This research was completed in two coastal plain watersheds in Maryland: the Choptank River watershed (U.S. Geological Survey [USGS] hydrologic unit code 02060005, ~1,756 km^2^) and the Corsica River watershed (USGS hydrologic unit code 020600020411, ~102 km^2^), a tributary to the Chester River (Figure 1). Both watersheds are dominated by agricultural land use (60%) with some forest (33% for the Choptank; 25% for the Corsica; [McCarty et al., 2008]). Twenty‐three forested wetland complexes across a 150 km^2^ area within the Choptank (*n* = 21) and the Corsica River (*n* = 2) watersheds were selected for this study. Most wetland catchments in this study (*n* = 14) were selected because of the ability to monitor the confluence between temporary and perennial streams at road crossings; the remaining catchments (*n* = 9) were located on state (Maryland Department of Natural Resources, DNR), conserved (The Nature Conservancy), or private lands with explicit permission from landowners.

Hydrology in this region, represented by seasonal and daily stream discharge patterns, is controlled by rainfall, temperature, evapotranspiration, topography, and soil drainage properties (Fisher et al., 2010). Annual precipitation (117 cm ± 4.2 cm; mean ± SE) is distributed uniformly throughout the water year (1986–2015 at Goldsboro, MD; PRISM climate mapping system [www.prism.oregonstate.edu]). Approximately 50% of annual precipitation is lost to the atmosphere via evapotranspiration while the remainder recharges groundwater or enters streams via surface runoff (Leahy & Martin, 1993). From approximately April to August, evapotranspiration and streamflow discharge rates exceed rainfall, leading to net water loss and falling groundwater levels (Fisher et al., 2010). Surface water levels typically reach peak expression in early spring (March/April) when levels of evapotranspiration are still relatively low (Lang et al., 2012). For the present study, historical monthly rainfall totals (1986–2015) were calculated using PRISM climate mapping system data (http://www.prism.oregonstate.edu/ downloaded on 13 October 2015; Supplemental Online, S2).

### 2.2 Catchment and stream delineations

**Figure 3:**
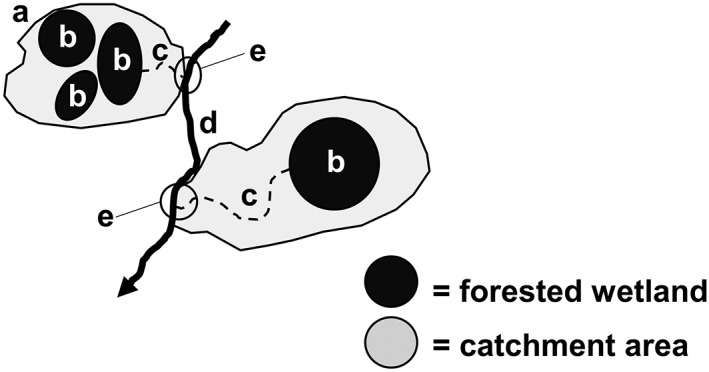
Schematic of forested wetland catchments, defined as relatively small areas of predominantly forested (generally, >50% forested) land (a) comprised of one or more seasonally inundated Delmarva bays (b) that produce episodic surface outflow into nonperennial streams (c), connecting them to the perennial stream network (d). Catchment outlets were defined as the nonperennial/perennial stream confluence (e)

Wetland catchments were defined as the total contributing area draining one or more forested Delmarva bay wetlands to a confluence with the perennial stream network. Resulting in part from human perturbations (e.g., ditching), most forested wetlands in the study area connect seasonally to the perennial stream network via surface flow (Figure 3). Catchment outlets (confluence points of temporary and perennial streams) were first identified within ArcGIS (ESRI; Redlands, CA) using a 2‐m digital elevation model (DEM) flow accumulation layer (D8 flow routing algorithm) to find contributing areas immediately upstream of the perennial stream network. A hand‐edited, flow accumulation‐based stream data set (Lang et al., 2012) was used to represent the perennial/intermittent stream network for the Choptank River watershed. The Lang et al. (2012) stream data set was designed to map streams that are fed by groundwater at least part of the year (perennial and intermittent) hydrology. Considering that the vast majority of streams identified by this method have surface water flow at least 9 months, we refer to these streams as “perennial.” For the present study, the Lang et al. (2012) methods were applied to the Corsica River watershed because the two wetlands in it extended beyond the spatial coverage of Lang et al.'s existing data layer (Supplemental Online, S1). Field visits with a handheld global positioning system (GPS) unit (Trimble Geo 7× model) were then conducted to validate catchment outlet locations and assess the eligibility of each site for long‐term monitoring. Sites were chosen based upon position within the same watershed (i.e., within the Choptank River or adjacent Corsica River watersheds), receipt of permission to work on the property, exhibition of characteristics indicative of Delmarva bays (e.g., elliptical shape, upland rim, alternating seasonal hydrology), and at least 50% forested cover.

## 3 METHODS

### 3.1 Hydrologic connectivity monitoring

Surface flow in temporary streams connecting forested wetlands to nearby perennial streams was recorded continuously over the 2015 water year (1 October 2014 to 30 September 2015). Float switch state data loggers (McDonough et al., 2015) were positioned in the thalweg of each temporary stream bed at the maximum longitudinal elevation along the channel to avoid local pools where standing water could falsely indicate the presence of surface flow. Surface water presence in maximum elevation areas within temporary streams was generally assumed to indicate a surface hydrologic connection between wetlands and nearby perennial streams, except in cases when a connection was not observed during site visits (e.g., short “flow” events recorded by state data loggers during summer precipitation events when wetlands were dry). Biweekly (November 2014–May 2015) or monthly (October 2014, June 2015–October 2015) site visits were made to validate or modify state data logger readings. At each site visit, discharge (L s^−1^) was measured using the cross‐sectional area method and a portable electromagnetic velocity metre.

For each catchment, state data logger records were used to generate three measures of connectivity over the water year: (a) *cumulative connection duration*, defined as the total number of days that wetlands connected to nearby perennial streams via surface flow; (b) *seasonal connection onset date*, defined as the Julian date of the first ≥24 hr connection event; and (c) *seasonal connection offset date*, defined as the Julian date of the last ≥24 hr connection event during the 2015 water year.

### 3.2 Landscape metrics

Winter's (Winter, 2001) hydrologic landscape conceptual framework informed our selection of landscape predictor metrics representing the hypothesized drivers of hydrologic connectivity, from field and GIS‐derived landscape variables generated at the reach and catchment scales (Table 1). This framework describes hydrologic landscapes based on land‐surface form, geology, and climate, which can be used to develop hypotheses of how the hydrologic system functions. This study used metrics characterizing land‐surface form and geology of catchments because climate conditions are similar across the sites. Metrics were classified into four groups: catchment, temporary stream, wetlands, and soils.

**Table 1:** Landscape predictor metrics tested against the wetland–stream surface hydrologic connectivity metrics and corresponding summary statistics

Indicator type	Predictor metric	Description	Mean (min, max) a
Catchment	CatchArea b	Catchment area (ha)	18.6 (1.0, 71.2)
	CatchSlope	Median catchment slope (m/m)	0.046 (0.027, 0.058)
	CatchRelief	Catchment relief (m)	4.2 (1.6, 7.5)
	HI	Hypsometric index (m/m)	0.43 (0.32, 0.58)
	CatchOutElev	Elevation at catchment outlet (m)	17.3 (14.1, 21.1)
	CatchShape	Catchment length:width ratio (dimensionless, m/m)	1.6 (1.0, 2.6)
	CatchVolStorage b ^,^ f	Catchment depressional surface storage volume (m^3^)	3,135.9 (48.0, 14,714.7)
	TWI c	Median topographic wetness index value in catchment	0.22 (0.20, 0.25)
	Dd b	Drainage density (m/m^2^)	1.9 (0.2, 5.7)
	Forest	Forest area (proportion of catchment)	0.79 (0.09, 1.00)
Temporary	StreamRelief d	Temporary stream relief (m)	0.02 (−0.67, 0.76)
Stream	StreamLength b ^,^ d	Temporary stream length (m)	79.2 (5.3, 386.6)
	StreamSlope d	Temporary stream slope (m/m)	−0.001 (−0.117, 0.007)
	BFW b ^,^ d	Temporary stream bankfull width (m)	1.99 (0.86, 4.5)
	BFD d	Temporary stream bankfull depth (m)	0.30 (0.05, 0.69)
	CSA b ^,^ d	Temporary stream cross‐sectional area (m^2^)	0.71 (0.05, 2.46)
	WDratio b ^,^ d	Temporary stream width:depth ratio (m/m)	7.56 (4.19, 18.3)
Wetlands	WetArea b	Wetland area (ha)	7.2 (0.2, 43.3)
	WetRelief b	Wetland spill relief threshold (m)	0.87 (0.38, 1.65)
	MeanWetDist f	Mean wetland‐to‐outlet distance (m)	209.9 (0, 628.7)
	MinWetDist f	Minimum wetland‐to‐outlet distance (m)	14.1 (0, 123.6)
	NumWet	No. wetlands (#)	6 (1, 16)
	WetInunScore b ^,^ g	Wetland hydrologic permanence score (numeric score, 1 to 6)	2.8 (2.0, 4.0)
Soils	Infildrained b ^,^ e	Soil infiltration rate, drained conditions (numeric score, 1 to 4)	1.98 (1.45, 3.09)
	Infilundrained e	Soil infiltration rate, undrained conditions (numeric score, 1 to 4)	3.20 (2.36, 3.99)
	WaterStorage e	Available water storage in from 0 to 150 cm soil depth (cm)	19.86 (16.34, 23.18)
	WTdepth e	Annual minimum water table depth (cm)	40.14 (6.05, 70.86)
	Ksat e	Saturated hydraulic conductivity (μm/se)	120.10 (24.62, 192.80)

a For ease of interpretability, mean, min, and max values were calculated prior to variable transformations.

b ln(x) transformed.

c 1/(x) transformed; TWI calculated as ln(a/tan β ), where “a” is upslope area per unit contour length and “tan β” is local slope in radians.

d Field‐derived.

e Area‐weighted mean.

f Normalized by catchment area for correlations and modelling procedures.

g Normalized by wetland area for correlations and modelling procedures.

Landscape predictor metric development and spatial analyses were conducted using ArcGIS (version 10.1; ESRI, Redlands, CA), R (version 3.2.2), and the Geospatial Modelling Environment (R‐Team, 2015; Beyer, 2012). GIS data layers were selected that (a) had spatial coverage across the Upper Choptank, Tuckahoe Creek, and eastern portion of the Corsica River watersheds (1,069 km^2^) useful for watershed‐wide SHC predictions; and (b) maximized our ability to detect fine‐scale variability between study catchments ranging in area from <1 to ≥70 ha.

### 3.3 Catchment metrics

Terrain analysis of high resolution digital elevation data was used to delineate flow paths, watersheds, and flow networks (after [Tarboton & Ames, 2001]; Supplemental Online, S3). Data were from a LiDAR based 2‐m DEM with a vertical accuracy ≤18 cm RMSE (Maryland DNR, 2003 and 2006 spring; http://dnrweb.dnr.state.md.us/gis/data/lidar/); these were designed to meet or exceed Federal Geographic Data Committee (Committee, 1988) National Standards for Spatial Data Accuracy for data at 1:2,400. Estimated horizontal positional accuracy of LiDAR point returns exceeds 50 cm. Bridges, roads, and other impediments to two‐dimensional flow were eliminated, and then bare earth LiDAR point data were rasterized to create a 2‐m resolution DEM using inverse weighted distance interpolation. Stream flow paths were then delineated from this corrected DEM using methods described below (Lang et al., 2012).

Catchment areas were calculated based on the D8 flow routing algorithm using the Terrain Analysis Using Digital Elevation Models software version 5.3 (Tarboton, 1997). Catchment outlets were defined as the highest flow accumulation cell upstream of a nonperennial/perennial stream confluence. Locations of the state data loggers in the temporary streams were field verified on 15 May 2015 using a handheld GPS (Trimble Geo 7× model), then snapped to the highest flow accumulation cell upstream of the temporary and perennial stream confluence. The Trimble Geo 7× GPS was designed to operate under a forest canopy and is capable of collecting data with submetre accuracy. GPS accuracy was enhanced by real‐time Wide Area Augmentation System correction and multiple (>15), GPS readings were collected at each location to increase the positional accuracy of the data.

*Catchment terrain slope* was calculated as the median slope (m/m) value within the catchment; slope direction was calculated using the Terrain Analysis Using Digital Elevation Models D‐Infinity flow routing algorithm. *Topographic wetness index* (TWI) was calculated for each catchment cell as ln(a/tan β), where “a” is the upslope area per unit contour length and “tan β” is the D‐Infinity slope (Beven & Kirkby, 1979). The median *TWI* value within the catchment was then used. Three elevation‐based metrics were calculated: *catchment relief* (the difference in elevation between the highest and lowest points in each catchment), *hypsometric index* (HI; an estimate of the relative distribution of elevation within each catchment; [Willgoose & Hancock, 1998]), and the *elevation at catchment outlet*. *Catchment shape* was defined as the catchment's length/width ratio (Bent & Steeves, 2006). *Catchment depressional storage volume*, an estimate of total surface depressional storage, was calculated by subtracting the bare earth DEM from the sink‐filled DEM, then summing these cell elevation differences across the catchment. *Drainage density* was defined as the channel length per unit catchment area and was calculated by manually digitizing channel lines in each catchment based on mapping stream channels with discernible bed and banks in the field using a handheld GPS (Trimble Geo 7× model), and using the 2‐m DEM and ancillary GIS layers (leaf‐off aerial imagery, flow accumulation raster) as reference. The Lang et al. (2012) stream data set was not used in calculating drainage density as it did not include ephemeral streams.

*Forest Area*, the areal percentage of forest land in each catchment, was calculated using the most recent state land use/land cover (LU/LC) data set available (MD Department of Planning 2010 data; metadata at: http://planning.maryland.gov/PDF/OurWork/LandUse/metadata.pdf; last accessed 05 May 2017). These data are based on digitization at the 1:12,000 scale using enhanced 2007 aerial imagery from the National Agriculture Imagery Program.

### 3.4 Temporary stream metrics

Because past studies have demonstrated that stream channel physical characteristics can serve as significant predictors of stream flow duration (Fritz, Johnson, & Walters, 2006; Svec, Kolka, & Stringer, 2005), temporary stream physical dimensions were measured at connectivity state data logger locations (04 September 2015) based on Fritz et al. (2006) methods. *Bankfull width* (BFW) and *bankfull depth* (BFD) were defined as the stream channel width and depth (at thalweg) at bankfull stage, respectively. Stream *cross‐sectional area* was calculated as BFW multiplied by BFD. Stream *width:depth ratio* was defined as the ratio of BFW to BFD.

Temporary stream *relief* (maximum elevation difference, using 2‐m DEM), *length* (flowpath distance between forested wetland spill point and catchment outlet), and *slope* (stream *relief/length*, using 2 m DEM) were calculated using stream lines GPS delineated in the field on 15 May 2015. Temporary stream lengths were delineated by walking upstream from each catchment outlet along the channel thalweg until the channel no longer had continuous defined bed and banks (Fritz et al., 2006). Field‐based estimates of temporary stream length were collected to compare the accuracy of field and GIS‐based delineations.

### 3.5 Wetland metrics

Past studies have demonstrated that wetland type and characteristics (e.g., size and number) influence surface hydrodynamics (McDonough et al., 2015; Shaw et al., 2013). The most recent state (MD DNR) wetland data set available was used to generate wetland metrics. (metadata at: http://imap.maryland.gov/ServicesMetadata/ImgBaseMapEarthCover/doqcir1m.htm; last accessed 06 May 2017). MD DNR developed this data set using the Cowardin, Carter, Golet, and LaRoe (1979) classification system and manual photo interpretation of aerial photographs (late 1980s–early 1990s) at the 1:12,000 scale.

**Figure 4:**
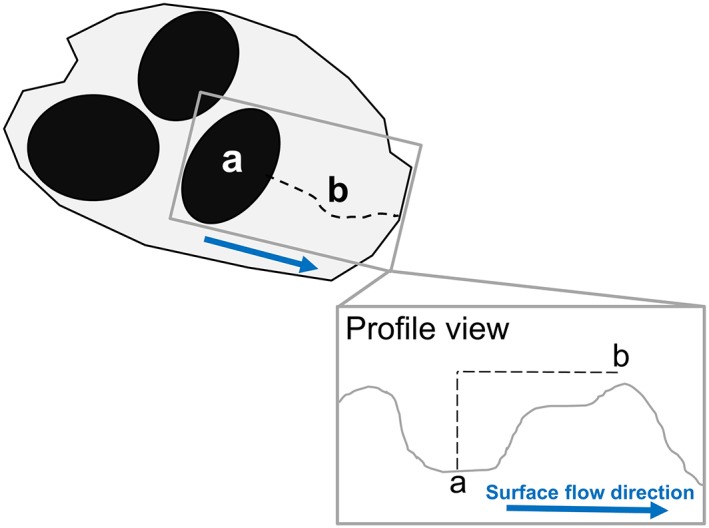
Wetland spill threshold relief was defined as the difference between the minimum elevation within the wetland (a) nearest to the catchment outlet (x), and the highest elevation along the nonperennial stream (b)

*Wetland area* was defined as the total wetland area (excluding farmed wetlands, [Cowardin et al., 1979], “Pf” classification) within each catchment. *Number of wetlands* was determined by the number of wetland polygons within each catchment. *Mean wetland distance* and *minimum wetland distance* were calculated by determining the Euclidean distance between each wetland polygon centroid and the catchment outlet. *Wetland spill threshold relief* was used to estimate the wetland surface water level needed to generate a surface hydrologic connection with the nearby perennial stream. It was calculated using the 2 m DEM as the difference in elevation between the highest point along the temporary stream and the lowest point within the wetland nearest to the catchment outlet (Figure 4).

The Cowardin et al. (1979) wetland classification includes a water regime modifier code, which describes hydrologic conditions during the growing season. Water regime values for wetlands within the study catchments ranged from saturated (substrate is saturated to the surface but typically no surface water present) to permanently flooded (water permanently covers the land surface; Cowardin et al., 1979). A *wetland hydrologic permanence score* was generated for each catchment by recoding wetland water regime values to a numerical scale from 1 (*saturated*) to 6 (*permanently flooded*), calculating an area‐weighted mean water regime value, normalized by total wetland area.

### 3.6 Soil metrics

Soil‐based metrics were generated using Soil Survey Geographic Database (SSURGO) soils data (version 2.2). SSURGO maps are created using manual photo interpretation at scales ranging from 1:12,000 to 1:63,630; minimum delineation size for Maryland surveys is approximately 0.6 ha. County‐level soils data were from the U.S. Department of Agriculture's Geospatial Data Gateway (https://gdg.sc.egov.usda.gov/; downloaded 18 August 2015) that were then clipped to the study area. Soil hydrologic groups range from “A” to “D,” with “A” soils having a very high infiltration rate (and hence a relatively low runoff potential) and “D” soils having a very low infiltration rate (and hence a relatively high runoff potential; NRCS, 2009). In some areas, soils are assigned a dual hydrologic group status (e.g., “A/D”) to indicate soil drainage properties in both “drained” (areas where seasonal high water table is kept at least 60 cm below the soil surface where it would be higher in a natural state) and “undrained” conditions, respectively. A catchment‐wide *infiltration score* was calculated using SSURGO data representing both drained (Infil_drained_) and undrained (Infil_undrained_) conditions. Hydrologic group values were assigned a numerical scale from 1 to 4 (*high to very low infiltration*), then aggregated to generate one area‐weighted mean catchment value.

*Available water storage* represented an estimate of the water volume that soil (0*–*150 cm depth) can store after having been wetted and free drainage has ceased; higher values are generally associated with low infiltration soil types (loams and clays). *Annual minimum water table depth* (WT_depth_) represented an estimate of the shallowest depth to a wet soil layer (water table) at any time during the year. *Saturated hydraulic conductivity* represented a soil's ability to transmit water when subjected to hydraulic gradient. As with *infiltration* score, all other soils‐based metrics were aggregated to generate an area‐weighted mean catchment value.

## 4 STATISTICAL ANALYSES

### 4.1 Effect of seasonality and precipitation on SHC

The null hypothesis that discharge (controlling for catchment area) in the temporary streams was independent of month and was tested with a one‐way ANOVA comparing log‐transformed monthly discharge. Discharge values were then permuted (i.e., for each catchment, values randomly reassigned to another month) 10,000 times; a one‐way ANOVA was calculated at each permutation and corresponding F‐statistic values were used to generate an F‐statistic distribution. The probability of observed discharge values under the null hypothesis was then assessed by calculating the proportion of permuted F‐statistic values greater than the observed F‐statistic value. Data processing was conducted using the “permute” package (version 0.8–4; Simpson et al. 2015) for R (version 3.2.2; R‐Team, 2015).

Paired Student's *t*‐tests (α = 0.05) were used to assess differences in mean 5‐day antecedent rainfall when SHC did and did not occur between the wetland and nearby perennial stream (after McDonough et al., 2015).

### 4.2 Relationship between SHC and landscape metrics

Preliminary exploration of the individual relationships between SHC metrics and landscape predictor metrics was conducted using Pearson's product moment correlation tests. Landscape predictor metrics that deviated substantially from normality based on the Shapiro–Wilk normality test were transformed (natural log or inverse of the metric). Spearman rank‐order correlation was used for heavily‐skewed predictor metrics. Strong correlations were noted as those with correlation coefficients greater than or equal to 0.40.

**Figure 5:**
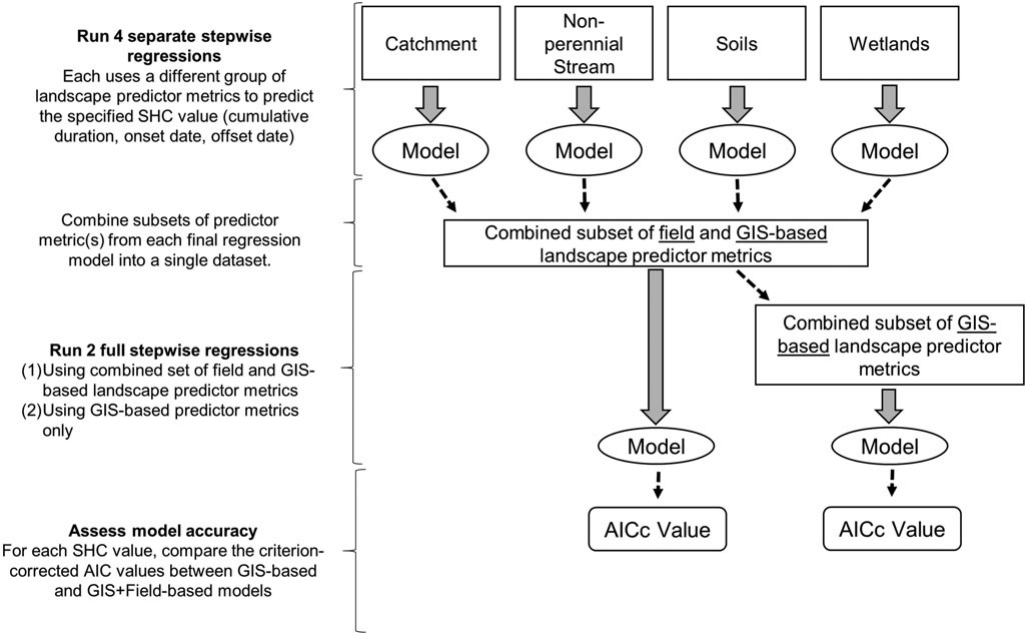
Stepwise regression procedure workflow

A forward stepwise linear regression approach (alpha‐to‐enter ≤0.05) was also used to model SHC patterns (cumulative connection duration, connection onset date, and connection offset date) as a function of the metrics. To reduce the number of metrics included (Austin & Steyerberg, 2015), separate stepwise regressions were first run using predictors from each of the four groups (catchment, temporary stream, soils, and wetlands). Significant predictors from these final regression models were then combined into a single data set to run a full, integrated stepwise regression with predictors from all four landscape predictor groups (Figure 5). Variance inflation cofactor values, which represent the degree to which variance of the estimated regression coefficients are inflated as compared to when the predictor metrics are not linearly related, were used to assess multi‐collinearity in final models (O'Brien, 2007). Landscape predictor metrics with Variance inflation cofactor less than 10 were included in final models.

Moran's I test was used to test for spatial autocorrelation in SHC metrics (cumulative connection duration, connection onset date, and connection offset date) across forested wetland catchments (Paradis, Claude, & Strimmer, 2004).

### 4.3 Comparing models with field versus GIS‐based metrics

To assess model improvement with the addition of field‐derived metrics, two stepwise regressions were run for each SHC metric: (a) using field and GIS‐derived landscape predictor metrics and (b) using only GIS‐derived metrics. Final regression models of each SHC metric were compared using the Akaike Information Criterion corrected (AICc) for small sample size. Additionally, AICc results were corroborated using a Fisher's *r*‐to‐*z* transformation and asymptotic *z*‐test to compare the correlation coefficients of observed versus predicted values between GIS + Field and GIS‐based regression models of each SHC value (Lee & Preacher, 2013).

The effect of field‐derived predictors on model accuracy was assessed by conducting two‐sample *t*‐tests to compare mean catchment characteristics (e.g., drainage density and BFW) between groups of catchments for which GIS‐based models overestimated (positive residuals) and underestimated (negative residuals) SHC values. All statistical analyses were conducted in R (version 3.2.2; R Development Core Team 2015).

## 5 RESULTS

### 5.1 Precipitation during the 2015 water year

Total rainfall during 2015 (133.7 cm) exceeded the 30‐year (1986–2015) average (117.4 cm ± 81.7 cm 95% CI); monthly rainfall totals were greater than the 30‐year normals during several months (November, December, January, March, and June) when wetland‐stream SHC is most likely to occur. Total 5‐day antecedent rainfall was significantly greater on days when a connection event occurred compared to 5‐day antecedent totals when a connection did not exist (*t* = 9.07, df = 22, *p* < .001, mean of differences = 5.40 mm).

### 5.2 Observed wetland‐stream connectivity patterns

**Figure 6:**
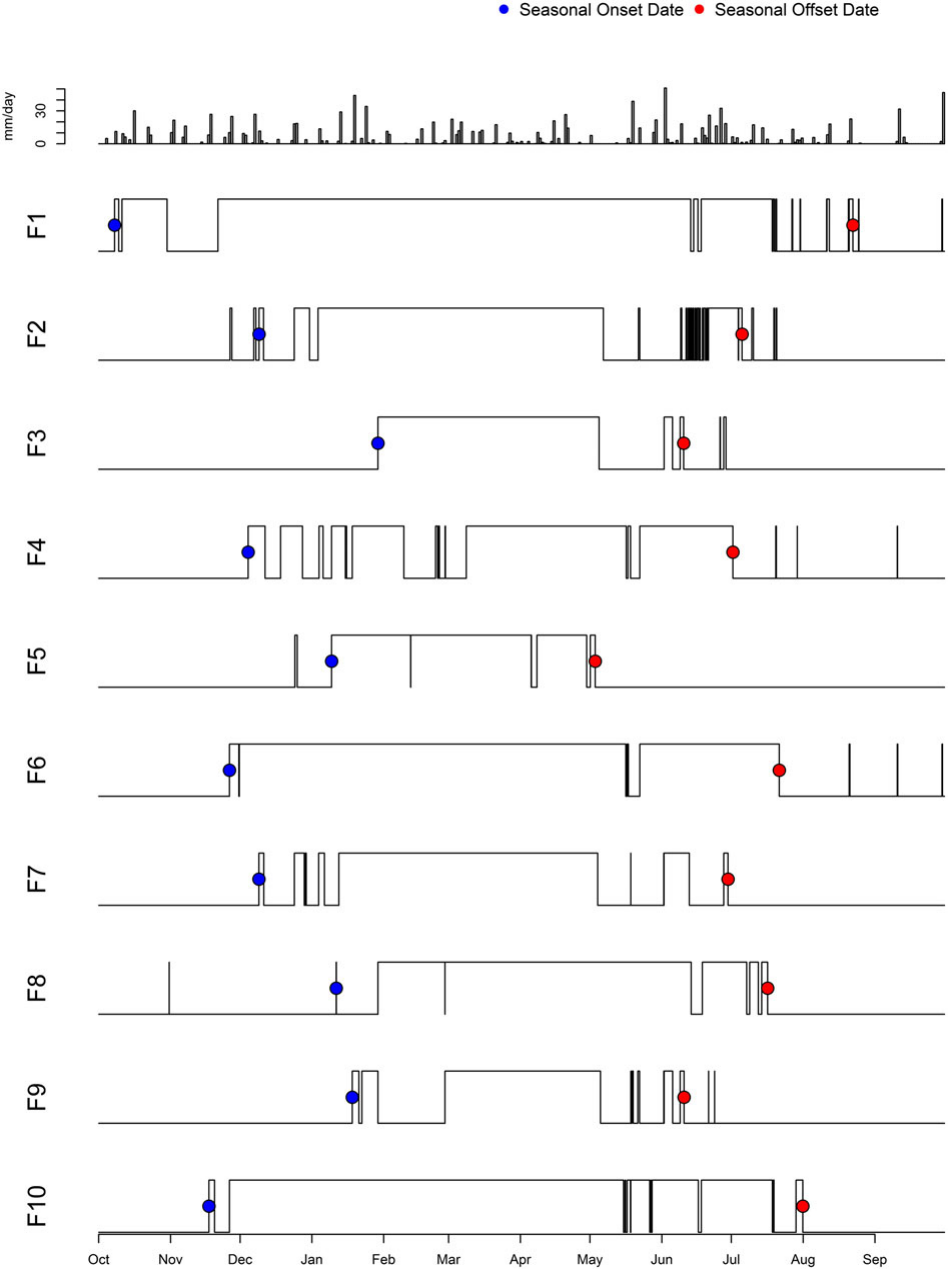
Daily rainfall totals (top panel) and surface hydrologic connectivity patterns for study catchments F1 to F10 during 2015 water year (1 October 2014 to 30 September 2015)

**Figure 7:**
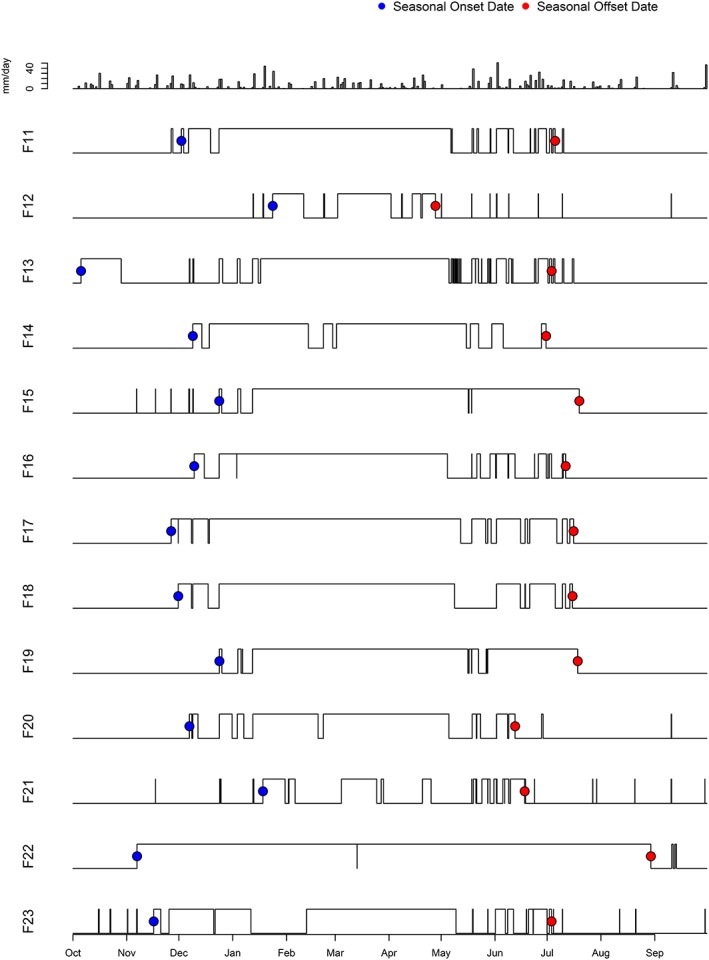
Daily rainfall totals (top panel) and surface hydrologic connectivity patterns for study catchments F11 to F23 during 2015 water year (1 October 2014 to 30 September 2015)

Surface flow patterns in temporary streams connecting forested wetlands to nearby perennial streams varied between wetland catchments (*n* = 23; cumulative wetland‐stream connectivity duration range 64–298 days; mean = 164.6 days). Between late‐spring and late‐fall, connections were short‐term (hours in duration) following rainfall events (Figures 6 and 7). Median seasonal connection onset and offset dates (first and last >24 hr connection) were December 9 and July 5, respectively (Table 2). Moran's I testing determined a lack of spatial autocorrelation in SHC metric values, including onset and offset dates, across forested wetland catchments.

**Table 2:** Forested wetland‐stream surface hydrologic connectivity metrics for 23 study catchments monitored for the 2015 water year (1 October 2014–30 September 2015)

Connectivity metric	Mean (SE)^a^	Median	Minimum	Maximum
Cumulative connection duration (d)	164.5 (12.3)	160.2	64.0	297.7
# connectivity transitions	13.7 (1.7)	11.0	5.0	36.0
Mean connection duration (d)	16.5 (2.5)	15.8	1.8	59.5
Max connection duration (d)	109.2 (9.6)	112.0	20.3	204.0
Seasonal onset connection date	December 9	December 9	October 5	January 29
Seasonal offset connection date	July 3	July 5	April 27	August 29

a SE = Standard Error.

**Figure 8:**
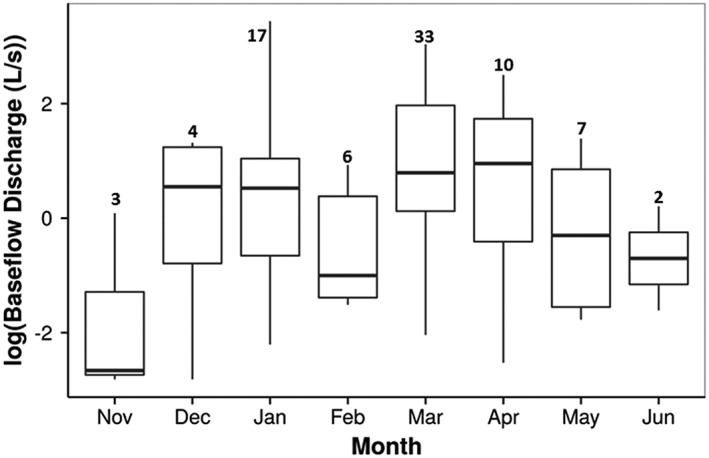
Boxplots of nonperennial stream baseflow discharge values (log transformed) collected each month during water year 2015. If measureable discharge was present, data were collected during biweekly visits (November 2014 to May 2015) and monthly (October 2014, June 2015–October 2015) visits. Text above boxplots indicates number of discharge measurements collected. Data were only collected during months shown due to no measureable discharge observed in remaining months (July–October)

Measurable baseflow discharge in temporary streams occurred November 2014–June 2015, during which time wetland surface water levels exceeded storage capacity, thus generating surface outflow to streams. Baseflow discharge from forested wetlands ranged from 0.06 to 31.19 L s^−1^ (0.002 to 1.1 ft^3^ s^−1^; Supplemental Online, T1). Overall differences in temporary baseflow among months were significant (F_7, 74_ = 2.56, *p* = .04); peak discharge occurred in early spring (March/April), during which surface water levels generally reach peak expression (Lang et al., 2012; Figure 8).

### 5.3 Landscape metrics as predictors of SHC

Cumulative connection duration was strongly correlated with landscape metrics (10 total) from all four predictor groups and primarily with wetland and temporary stream metrics: *ln‐wetland area* (*r* = 0.65), *number wetlands* (*r* = 0.63), *ln‐temporary stream channel BFW* (*r* = 0.60,), *ln‐wetland hydrologic permanence score* (*r* = −0.60), and *ln‐catchment area* (*r* = 0.55; Table 3).

**Table 3:** Pearson's product moment correlation between surface hydrologic connectivity metrics and landscape predictor metrics

Landscape predictor group	Predictor metric	Cumulative connection duration (d)	Seasonal connection onset date	Seasonal connection offset date
Catchment	CatchArea a	**0.55** ^*^	−0.28	**0.64** ^*^
	CatchSlope	−0.15	**0.46** ^*^	−0.13
	CatchRelief	0.36	−0.16	0.53
	HI	−0.24	0.11	−0.23
	CatchOutElev	−0.01	−0.27	−0.08
	CatchShape	0.18	−0.03	0.25
	CatchVolStorage a ^,^ e	0.16	<0.01	0.21
	TWI b	−0.10	0.52*	−0.18
	Dd a	0.25	**−0.52** ^*^	0.24
	Forest g	0.21	0.13	0.19
Temporary stream	StreamRelief c	0.20	−0.12	0.26
	StreamLength a ^,^ c	0.44*	−0.42*	0.50*
	StreamSlope c ^,^ g	0.23	−0.16	0.16
	BFW a ^,^ c	0.60*	−0.49*	0.53*
	BFD c	0.49*	−0.43*	0.35
	CSA a ^,^ c	0.54*	−0.47*	0.45*
	WDratio a ^,^ c	−0.18	0.23	−0.05
Wetlands	WetArea a	0.65*	−0.35	0.68
	WetRelief a	0.28	−0.31	0.28
	MeanWetDist e	−0.51*	0.39	−0.33
	MinWetDist e	−0.37	0.34	−0.48*
	NumWet	0.63*	−0.35	0.65*
	WetInunScore a ^,^ f	−0.60*	0.35	−0.63*
Soils	Infildrained a ^,^ d	0.25	−0.27	0.11
	Infilundrained d	0.25	−0.04	0.01
	WaterStorage d	0.47*	−0.44*	0.33
	WTdepth d	−0.05	−0.21	0.06
	Ksat d	−0.21	0.32	−0.29

*Note*. Correlation coefficients ≥0.40 denoted by bold and *. See Table 1 for explanation of landscape predictor metric abbreviations. BFW = bankfull depth; BFD = bankfull width; CSA = cross‐sectional area; TWI = topographic wetness index; WDratio = width:depth ratio

a ln(x) transformed;

b 1/(x) transformed.

c Field‐derived.

d Area‐weighted mean.

e Normalized by catchment area.

f Normalized by wetland area.

g Spearman rank correlation conducted due to heavily skewed predictor metric distribution.

The landscape predictor metrics most strongly correlated with seasonal connection onset date were *inverse‐median TWI value* (*r* = 0.52), *ln‐drainage density* (*r* = −0.52), *ln‐temporary stream BFW* (*r* = −0.49), *ln‐temporary stream cross‐sectional area* (*r* = −0.47), and *median catchment slope* (*r* = 0.46; Table 3). The following predictor metrics were most strongly correlated with prolonged (>24 hr) SHC events that occurred later in the 2015 water year (i.e., seasonal connection offset date): *ln‐wetland area* (*r* = 0.68), *number wetlands* (*r* = 0.65), *ln‐catchment area* (*r* = 0.64,), and *ln‐wetland hydrologic permanence score* (*r* = −0.63; Table 3).

Three temporary stream metrics were strongly correlated with all three SHC metrics: *temporary stream length*, *temporary stream BFW*, and *temporary stream cross‐sectional area* (Table 3). Longer, deeper channels were associated with more prolonged periods of surface flow that initiated earlier and remained longer through the water year.

Models built as a function of both field and GIS‐derived predictor metrics explained the most variability in cumulative connection duration (Adj. *R* ^2^ = 0.80), followed by seasonal connection onset date (Adj. *R* ^2^ = 0.69) and seasonal connection offset date (Adj. *R* ^2^ = 0.53; Table 4). However, the AICc results indicated that model accuracy was not significantly improved by the addition of field‐derived predictors. For each SHC metric, the removal of field‐derived predictor metrics from stepwise regression resulted in final GIS‐based models with ∆AICc values of −4.0 (connection duration), −0.2 (seasonal connection onset date), and 2.1 (seasonal connection offset date) compared to the model based on both field and GIS‐derived predictor metrics. Models with a ∆AICc value greater than 3 are generally considered to have considerably less support than the minimum AICc model for a given data set (Burnham & Anderson, 2002). Using this threshold suggests no need to retain field‐derived predictor metrics in the models. The asymptotic *z*‐test comparing the correlation coefficients of observed versus predicted values between the GIS + Field and GIS‐based corroborated this result, as indicated by non‐significant differences in the observed versus predicted correlation strengths.

**Table 4:** Comparison of stepwise regression models developed for SHC metrics using (a) GIS‐based predictor metrics and (b) field and GIS‐based metrics

Response variable	Predictor groups used in model	Final model	Model AICc value	Model adjusted *R* ^2^ value	Model observed vs. predicted values
Cumulative SHC duration (days)	Field and GIS	= −190.6−31.3 (CatchShape) + 10.7 (Dd) + 79.6 (Forest) + 61.9 (BFW) + 15.0 (WetArea) + 3.0 (NumWet) + 13.3 (WaterStorage)	237.5	0.80, *p* < .0001	*r* = 0.92, *p* < .0001
	GIS	= −260.5 + 42.4 (WetArea) + 11.1 (NumWet) + 21.0 (WaterStorage)−51.2 (CatchArea)	233.5	0.74, *p* < .0001	*r* = 0.89, *p* < .0001
Connection onset date (Julian date)	Field and GIS	= −267.7−11.6 (CatchArea) + 44.5 (CatchShape) + 79.6 (TWI)−14.6 (Dd)−77.3 (Forest)−22.6 (BFW) + 16.0 (WetRelief)	218.0	0.69, *p* = .0004	*r* = 0.89, *p* < .0001
	GIS	= −272.7 + 24.1 (CatchShape) + 99.3 (TWI)−76.3 (Forest)−2.9 (NumWet)−4.0 (WaterStorage)	217.8	0.58, *p* = .001	*r* = 0.82, *p* < .0001
Connection offset date (Julian date)	Field and GIS	= 244.5 + 24.0 (BFW) + 12.9 (WetArea)	209.1	0.53, *p* = .0002	*r* = 0.76, *p* < .0001
	GIS	= 255.7 + 15.4 (WetArea)	211.3	0.44, *p* = .0004	*r* = 0.68, *p* = .0004

*Note*. Models built using full data set (*n* = 23); full predictor metric names and descriptions in Table 1. BFW = bankfull depth; TWI = topographic wetness index.

## 6 DISCUSSION

Using a field‐validated, landscape‐scale approach to quantify Delmarva bay wetland‐stream SHC, this study demonstrates that field and GIS‐derived metrics can be used to explain and predict variability in wetland‐stream connectivity at the landscape scale. By modelling connectivity metrics as a function of catchment, wetland, temporary stream, and soil characteristics representing hypothesized SHC drivers, these results contribute to the new field of research aimed at developing relatively low‐cost, scalable approaches for quantifying flow permanence throughout stream networks (Bhamjee, Lindsay, & Cockburn, 2016) and wetland landscapes. Combining rainfall data with continuous measurements of SHC between wetlands and temporary streams, this study provides evidence of seasonal changes in the underlying drivers of patterns in connectivity. The combination of GIS data and extensive field data from 23 wetland‐stream sites allows us to narrow the suite of landscape factors influencing the timing and duration of wetland‐stream SHC. An important next step is to field‐test predictions of the connectivity model developed in this study and, as in (Golden et al., 2016), scale‐up such studies to understand the cumulative effect of wetlands on broader waterways.

### 6.1 Temporal variability in wetland‐stream connectivity patterns

Delmarva bays are complex systems whose degree of landscape connectivity is a function of both local and regional hydrological processes. Like other depressional wetlands, SHC between Delmarva bays and streams is a function of water balance within wetland catchments and landscape attributes including soils and perhaps size (Golden et al., 2016; Leibowitz & Nadeau, 2003; Sharitz, 2003). Connections are most likely to occur during periods when water inputs (precipitation, groundwater discharge) exceed water losses (evapotranspiration, groundwater recharge; Lide, Meentemeyer, Pinder, & Beatty, 1995; Sharitz, 2003), leading to surface outflow from wetlands into nearby streams. The results from this 2015 field study indicate that, as was the case in the 2010 water year in this watershed (McDonough et al., 2015), seasonal groundwater dynamics drive the timing of prolonged (≥ 24 hr) surface water connections between forested Delmarva bay wetlands and perennial streams from late fall to late spring. During the 2015 water year, more than half of wetland‐stream surface connections turned “on” and “off” within a 3‐week period (Figures 6 and 7). The spatiotemporal homogeneity of SHC onset and offset dates and absence of spatial autocorrelation across the study area suggests that a seasonal drop in evapotranspiration, followed by a regional rise in groundwater table, exert first‐order controls over sustained outflow of surface water ponding within bays to temporary streams when the water table is at or above the surface (Lide et al., 1995).

The shortened (minutes to hours), duration of SHC events observed between late spring and late fall in 2015 reflects a seasonal shift in the drivers of SHC. These shortened SHC events coincided with the seasonal peaks in transpiration, during which Delmarva bays typically lack surface water (Fisher et al., 2010; Phillips & Shedlock, 1993), and generally represented ephemeral surface water levels in temporary streams following rain events. Over the study year, recent rainfall amounts, as indicated by 5‐day antecedent rainfall totals, were significantly higher on days when a SHC occurred compared to non‐SHC days, indicating that antecedent conditions and local fill‐spill dynamics also influence wetland‐stream connectivity in Delmarva bays.

The delay from SHC onset to measureable baseflow discharge in temporary streams suggests a mechanistic shift in the SHC driver from groundwater (i.e., ponded surface water level with the groundwater table in the surrounding soils and wetland) to surface water outflow (i.e., wetland spillage) from fall to winter. Although the median seasonal onset date occurred on December 9, baseflow discharge was not measureable (i.e., water depth ≤ 3 cm and/or no measureable water velocity in channel) in most catchments until late January (Supplemental Online, Table T1). Field observations confirm that this shift was aligned with bay ponding levels exceeding their relative spill thresholds and flowing into the adjacent temporary streams (Figure 4). This finding is consistent with the model of wetland connectivity described by (Winter & LaBaugh, 2003), in which surface outflow is described as a function of groundwater flow, spill elevation above normal wetland water level, and the timing of precipitation events.

**Figure 9:**
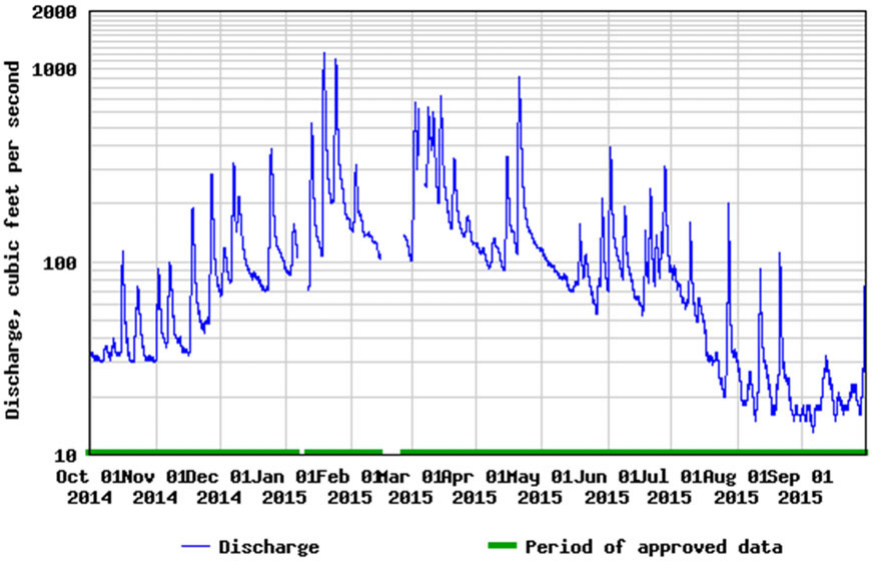
Stream discharge (cfs) record for Tuckahoe Creek near Ruthsburg, MD (USGS station 01491500) for the 2015 water year. Data and graphic acquired from the USGS WaterWatch web portal (accessed 8 Jan 2016)

The effects of seasonal shifts in surface and groundwater dynamics on hydrology at the landscape scale are evident in the seasonal increase in baseflow discharge in Tuckahoe Creek, a tributary to the Choptank River, during the winter and early spring (Figure 9). It is well‐documented that depressional wetlands, in aggregate, have a substantial effect on watershed‐scale water balances by increasing seasonally defined subsurface storage and groundwater flow (Evenson, Golden, Lane, & D'Amico, 2015). During the 2015 water year, peak temporary stream baseflow discharge below forested wetlands was observed in early spring, indicating that Delmarva bay wetlands contribute to mainstem river discharge via direct surface outflow at least seasonally. This finding is consistent with (Golden et al., 2016) which reports a seasonal effect on the relationship between wetland characteristics and streamflow in the Middle Atlantic Coastal Plain ecoregion (North Carolina, United States), including a significant relationship between wetland area and streamflow during the spring, when poorly drained wetland systems respond rapidly to precipitation events.

Regressions in the current study indicated that one or of both the wetland area and wetland number metrics was related to SHC duration and seasonal offset date (Table 3). Future studies are needed to quantify the partitioned (surface water vs. groundwater) and/or aggregate effect of wetland‐stream connectivity on downstream waters (e.g., mean seasonal increase in mainstem river baseflow during connections). In addition to linking SHC patterns to downstream ecological processes, future studies should investigate the relationship between wetland‐stream groundwater hydrologic connectivity and landscape characteristics (e.g., bay size and soil type; [McLaughlin, Kaplan, & Cohen, 2014]) to better quantify the downstream effects of such connections.

### 6.2 Landscape characteristics as predictors of wetland‐stream connectivity patterns

Among the landscape predictor metrics, temporary stream length, BFW, and cross‐sectional area were strongly correlated with all three SHC metrics, and BFW was a significant predictor in all final regression models. Several studies have reported these physical measurements to be significant predictors of stream flow duration, including BFW (Fritz, Winerick, & Kostich, 2013; Svec et al., 2005) and entrenchment ratio (flood prone width divided by BFW; [Fritz, Johnson, & Walters, 2008; Svec et al., 2005]), though Fritz et al. (2013) caution they may be weak predictors in high rainfall, low topographic relief regions with low erosive potential. Although correlations between metrics do not imply causation, two mechanisms may explain the relationships between SHC metrics and temporary stream channel geomorphology. First, surface water presence in temporary stream channels may be an expression of the shallow regional depth to groundwater in our study region (the minimum‐recorded depth at a nearby well during the 2015‐water year was 0.86 m) with deeper and wider channels perhaps more suggestive of prolonged flow, including during periods before and after wetland‐stream SHC. Second, larger stream channels may be evidence of the effect of higher flows from wetland surface outflow on maintaining or actively shaping channels. In their study of flow duration in headwater streams throughout South Carolina Piedmont and Southeastern Plains, where catchment relief and stream discharge values were similar to this study, Fritz et al. (2013) cite channel geomorphology as an important parameter in discriminating headwater stream flow class.

In general, larger, wetter catchments (greater number and area of wetlands, higher wetland hydrologic permanence score) were associated with greater cumulative SHC duration and later SHC offset dates. These results agree with earlier findings that wetland area (McDonough et al., 2015) and total catchment area (Lampo, 2015) are positively related to headwater stream flow duration in flat, well‐drained landscapes. Larger, wetter catchments were also associated with larger temporary stream channels, illustrating the potential effect of collinearity among landscape predictor metrics in accurately identifying drivers of observed SHC patterns (Supplemental Online Figure F1 **)**. Future studies should consider paired‐sensor approaches to discriminate between periods of ponding (i.e., groundwater‐fed) and streamflow (i.e., wetland surface outflow) in temporary streams connecting wetlands and streams (Bhamjee et al., 2016), which may help better link landscape characteristics to hydrological patterns.

### 6.3 Evaluating the accuracy of landscape predictor‐based regression models

Stepwise regression has been applied in several other studies within the mid‐Atlantic Coastal Plain region of the Unite States to model hydrological patterns as a function of landscape characteristics (Julian, Elmore, & Guinn, 2012; McDonough et al., 2015). Applying a similar technique in this study provides an opportunity to compare findings. Based on final models and non‐significant differences in observed versus predicted correlation strengths between models that included GIS + Field metrics and models with only GIS, the addition of field‐derived predictor metrics did not significantly improve model performance (Table 4). In fact, the removal of field‐derived predictor metrics from stepwise regression led to a final GIS‐based model of cumulative SHC duration with more support, as indicated by a decrease in AICc value of 4.2 (Burnham & Anderson, 2002). These results suggest that among the variables used in this study, GIS + Field and GIS‐based models performed comparably in their ability to explain variability in SHC patterns among forested Delmarva bay wetland catchments.

In their study linking landscape attributes to channel head locations, Julian et al. (2012) concluded that the occurrence of channel heads across Maryland's Coastal Plain was most likely driven by saturation overland flow given the sandy soils and close proximity of the water table. Further, they note that sorted bedload and definable banks were often evident several metres downstream of wetlands. Our results support this conclusion, as GIS‐based indicators of contributing area (CatchArea), wetland extent (WetArea, NumWet), and saturation overland flow potential (WaterStorage) were included in final models of surface flow duration and seasonal connection onset date in headwater streams (Table 1). Contributing area, which can be readily calculated using DEM analysis techniques, has been consistently reported as a significant predictor of flow permanence in a range of geographic settings (Bent & Steeves, 2006; Fritz et al., 2013; Montgomery & Dietrich, 1988).

## 7 CONCLUSIONS

Recent emphasis has been placed on the need for research on the frequency, magnitude, timing, and duration of flows from wetlands that are traditionally considered to be geographically isolated and temporary streams to down‐gradient waters (Acuña et al., 2014; Rains et al., 2016). Worldwide, there are still gaps in our scientific understanding of the diverse roles that geographically isolated wetlands and temporary channels play in the hydrology and ecology of nearby waters (Costigan, Jaeger, Goss, Fritz, & Goebel, 2016; Datry, Pella, Leigh, Bonada, & Hugueny, 2016; McLaughlin & Cohen, 2014; Vanderhoof et al., 2017). To our knowledge, this present study is one of only two (McDonough et al., 2015) that provide a robust, field‐based data set on the surface hydrological connectivity patterns between forested Delmarva bay wetlands and perennial streams. Among landscape predictor metrics, variability in connectivity was most strongly explained by *catchment area*; *wetland area*, *number*, and *mean wetland hydrologic permanence score*; and *temporary stream channel dimensions*. Larger, wetter catchments with deeper temporary stream channels were associated with greater cumulative SHC duration and later seasonal connection offset dates.

Linking hydrologic dynamics to landscape structure provides an important foundation for GIS‐based analysis of environmental drivers of SHC. Our finding that the addition of field‐derived data did not improve predictive models suggests that GIS‐derived metrics alone may be adequate for predicting multiple aspects of SHC. Recent technological improvements in the quality and spatial resolution of remote sensing and GIS products provide increasing opportunities to accurately model hydrological patterns as a function of GIS‐based variables. High‐resolution (≤5 m) satellite imagery is currently available upon request (Vanderhoof, Alexander, & Todd, 2016; Vanderhoof, Alexander, & Todd, 2017) and will become available at near‐daily recurrence intervals in the near future (Tiner, Lang, & Kleman, 2015). The coverage of high‐resolution data is increasing as well. In the Unites States, for example, future versions of the National Hydrography Data set will incorporate national, high‐resolution elevation data (Viger, Rea, Simley, & Hanson, 2016) from the 3D Elevation Program (Sugarbaker et al., 2017). The 3D Elevation Program, a partnership led by the U.S. Geological Survey, is systematically collecting and processing enhanced elevation data for all U.S. states and territories. Upon completion, anticipated in 2023, products and source data for the entire United States will be freely available online (Sugarbaker et al., 2017). These and other efforts, for example, the Open Geospatial Consortium (OCG, 2017) to provide increased access to high‐quality, high‐resolution, attribute‐rich geospatial data sets are establishing new standards of precision and accuracy for analyses of bare‐earth and above‐ground features in natural and human‐altered landscapes.

Given the role of seasonal groundwater table dynamics as a driver of wetland‐stream connectivity in the broader region (Lide et al., 1995), our results likely reflect broad SHC patterns in forested wetlands across the Delmarva bay landscape and may be directly applicable to depressional bays across the U.S. Mid‐Atlantic and Southeastern Coastal Plain, where climate and landscape controls of hydrological inputs and losses are expected to be similar to our study area. The SHC patterns and their relationships with predictor metrics in our study area will be most directly applicable to shallow, seasonal wetland complexes that are both precipitation and groundwater driven. As wetlands fill with seasonal precipitation and regional groundwater table rises, inundated wetlands reach a saturation threshold at which they merge with co‐located wetlands and spill into defined, temporary flowpaths. In our study system, the outlets of wetland catchments were situated within a relatively short distance (e.g., <400 m) from perennial stream networks. Our results highlight the importance of local and regional surface water‐groundwater interactions, wetland density, wetland area, and characteristics of the connecting flowpath on the onset and duration of wetland outflow to stream networks.

Our results can also broadly inform research on watershed connectivity by comparison to studies in contrasting landscapes or studies conducted at different spatial or temporal scales. For example, Vanderhoof et al. (2016, Vanderhoof, Distler, et al., 2017) used Landsat imagery to quantify landscape‐scale connectivity in the Prairie Pothole Region. They demonstrated that connectivity via merging of wetlands and wetlands with streams was positively related to total wetland area (also reported by Kahara, Mockler, Higgins, Chipps, & Johnson, 2009) but sensitive to the definition of the wet–dry threshold used to classify Landsat pixels or subpixels as “water” or “land” (Vanderhoof et al., 2016). Temporal and spatial scales of analysis such as those in Vanderhoof et al. (2016) are not achievable using static data sets or most field studies. However, as the authors acknowledge, the resolution of Landsat imagery (30 m) is biased against detection of connectivity via small features, such as those measured here. Smaller scale studies, in turn, may miss variation in drivers of connectivity that occur over longer timeframes or over larger spatial scales. Integration of data sets from different sources to improve accuracy and resolution and modelling over larger spatial and temporal scales is an emerging science (DeVries, Pratihast, Verbesselt, Kooistra, & Herold, 2016; Vanderhoof et al., 2017). However, trade‐offs between model fidelity and complexity, as well as scale, and interpretation of differences in the climate and landscape that drive dominant connectivity mechanisms (e.g., expansion and merging of surface waters vs. precipitation/groundwater driven fill‐and‐spill) are required to determine the appropriate application of a model (Golden et al., 2017), including the one presented here.

Lastly, this study can inform stream and wetland restoration efforts in the Delmarva Peninsula. The wetland complexes in this study are all seminatural and are hydrologically connected to perennial waterways via small, intermittent channels (including some historical ditches that are no longer maintained and are structurally indistinguishable from natural streams). Across the Peninsula, approximately 70% of Delmarva bays have been hydrologically altered for agriculture (Fenstermacher, Rabenhorst, Lang, McCarty, & Needelman, 2014), typically by ditch drainage, which increases transport of water and contaminants (e.g., nitrogen) to streams and the Chesapeake Bay (Denver et al., 2004). In a recent study of potential surface water storage, a key landscape function through which Delmarva bays retain and process constituents of runoff, Jones et al. (2017) estimated that plugging of ditches would increase natural water storage capacity by 76% across the entire Delmarva Peninsula, and 250% in a single subcatchment, roughly equivalent to a HUC12, that has a high density of Delmarva bays. Full restoration of landscape functions of Delmarva bay wetlands is dependent upon a better understanding of the factors controlling their hydrologic connectivity to streams (McDonough et al., 2015). This study helps fill gaps in our understanding of the landscape and climate factors that control the hydrodynamics of Delmarva bays and temporary coastal plain streams, thereby improving landscape approaches to the quantification of the surface hydrologic connections by which so‐called geographically isolated wetlands contribute to the integrity of perennial waters.

## Supporting information

Data S1. Supporting Information
**Table T1.** Water year 2015 temporary stream baseflow discharge measurements. ‐‐‐ = site not visited, * = non‐continuous surface flow present,! = continuous surface flow present, but no measurement taken
**Figure F1.** Correlation matrix of landscape predictor metrics, where circle colour and size represent the strength and direction (positive or negative) of the correlation between each predictor metric pair. See Table 1 for explanation of landscape predictor metrics.
