# Supplementary material for: Landscape metrics as predictors of hydrologic connectivity between Coastal Plain forested wetlands and streams
Source: Hydrol Process. 2018 Feb 20;32(4):516–32. doi: 10.1002/hyp.11433 (PMC5856080; doi:10.1002/hyp.11433)
Supplement: Supplementary file 1 — Data S1. Supporting Information Table T1. Water year 2015 temporary stream baseflow discharge measurements. ‐‐‐ = site not visited, * = non‐continuous surface flow present,! = continuous surface flow present, but no measurement taken Figure F1. Correlation matrix of landscape predictor metrics, where circle colour and size represent the strength and direction (positive or negative) of the correlation between each predictor metric pair. See Table 1 for explanation of landscape predictor metrics. [file HYP-32-516-s001.docx]

**fSupplemental Online Material**

**S1**. Briefly, the Lang et al. (Lang, McDonough, McCarty, Oesterling, & Wilen, 2012) method included using ArcGIS ArcHydro (ESRI; Redlands, CA) tools to automatically delineate stream networks at a flow accumulation threshold of 30 ha then hand-editing stream networks using several recent leaf-on and leaf-off aerial images to include only streams that met a minimum set of criteria (e.g., water appeared to be present within the channel within the last decade, a vegetation buffer was present around the channel). The resulting stream datasets include only streams judged to be perennial or intermittent and therefore groundwater fed at some point during a year of normal precipitation.

**S2.** The climate data were obtained from stations (13,000 stations across the conterminous U.S.) and weighted to control for the effects of additional variables, such as proximity to nearby stations, topographic position, and coastal proximity (Daly et al., 2008)). For this study PRISM 4km grid data were used to estimate daily rainfall totals at each forested wetland catchment during the 2015 water year.

**S3.** DEM sinks (cells completely surrounded by higher elevation cells) were filled to create a depressionless DEM, flow direction assigned to each grid cell based on the direction(s) of steepest elevation descent, and flow accumulation calculated across the DEM, where cell values denoted the number of upslope cells flowing into that cell. A flow accumulation threshold was then applied to define stream channels, and catchments are delineated by identifying all grid cells contributing surface flow to a given outlet point.

**Table T1.** Water year 2015 temporary stream baseflow discharge measurements. --- = site not visited, * = non-continuous surface flow present, ! = continuous surface flow present, but no measurement taken

| Baseflow Discharge (L s^-1^) | | | | | | | | | | | | | | |
| --- | --- | --- | --- | --- | --- | --- | --- | --- | --- | --- | --- | --- | --- | --- |
|  | Site | 11/30/2014 | 12/18/2014 | 1/09/2015 | 1/11/2015 | 1/29/2015 | 2/12/2015 | 3/12/2015 | 3/13/2015 | 3/29/2015 | 4/16/2015 | 5/05/2015 | 6/25/2015 |  |
|  | F1 | 1.09 | 3.73 | --- | 0.46 | --- | --- | --- | 11.44 | 5.55 | 6.43 | 2.11 | --- |  |
|  | F2 | * | * | --- | ! | --- | --- | --- | 7.14 | 2.10 | 2.77 | 0.17 | --- |  |
|  | F3 | * | * | * | --- | 0.11 | NA | --- | 1.45 | 0.79 | 0.63 | * | --- |  |
|  | F4 | * | ! | 0.19 | --- | 2.12 | --- | --- | 7.16 | 7.85 | 3.90 | 0.25 | --- |  |
|  | F5 | * | * | ! | --- | 0.52 | --- | --- | 1.04 | 0.21 | 0.78 | * | --- |  |
|  | F6 | 0.06 | 3.37 | 2.83 | --- | 31.19 | --- | --- | 20.78 | 11.98 | 12.19 | 2.61 | --- |  |
|  | F7 | --- | --- | * | --- | --- | 0.25 | 1.71 | --- | --- | --- | --- | --- |  |
|  | F8 | * | * | --- | ! | 8.33 | --- | 12.38 | --- | 11.44 | --- | 4.01 | --- |  |
|  | F9 | * | * | --- | * | * | --- | 4.01 | --- | 1.62 | --- | * | --- |  |
|  | F10 | --- | --- | --- | --- | 1.69 | 0.25 | 1.13 | --- | --- | --- | --- | --- |  |
|  | F11 | --- | --- | --- | --- | 1.12 | 0.22 | 0.99 | --- | --- | --- | --- | --- |  |
|  | F12 | --- | --- | --- | --- | 0.39 | * | 0.25 | --- | --- | --- | --- | --- |  |
|  | F13 | * | * | * | --- | --- | --- | --- | ! | 0.13 | 0.08 | * | --- |  |
|  | F14 | --- | 0.06 | ! | --- | 4.43 | --- | --- | 2.00 | ! | --- | ! | * |  |
|  | F15 | --- | --- | --- | * | --- | 2.04 | 2.21 | --- | --- | --- | --- | --- |  |
|  | F16 | --- | --- | --- | ! | --- | 2.53 | 8.82 | --- | --- | --- | --- | --- |  |
|  | F17 | 0.07 | 0.89 | 1.58 | --- | 5.93 | --- | --- | 10.60 | 7.04 | 10.48 | 0.74 | 1.23 |  |
|  | F18 | * | * | 0.57 | --- | 2.58 | --- | --- | 3.87 | 3.26 | 2.43 | 0.18 | 0.20 |  |
|  | F19 | --- | * | * | --- | 2.70 | --- | --- | 3.70 | 1.04 | --- | * | * |  |
|  | F20 | --- | * | --- | --- | ! | --- | --- | 1.18 | * | --- | * | * |  |
|  | F21 | --- | --- | --- | --- | --- | --- | --- | 0.16 | --- | --- | --- | --- |  |
|  | F22 | --- | --- | --- | --- | --- | --- | --- | ! | --- | --- | --- | --- |  |
|  | F23 | --- | --- | --- | ! | --- | 0.54 | --- | 2.05 | --- | 0.59 | --- | --- |  |
|  | Mean | 0.41 | 2.01 | 1.29 | 0.46 | 5.09 | 0.97 | 3.94 | 5.58 | 4.42 | 4.03 | 1.44 | 0.72 |  |

**
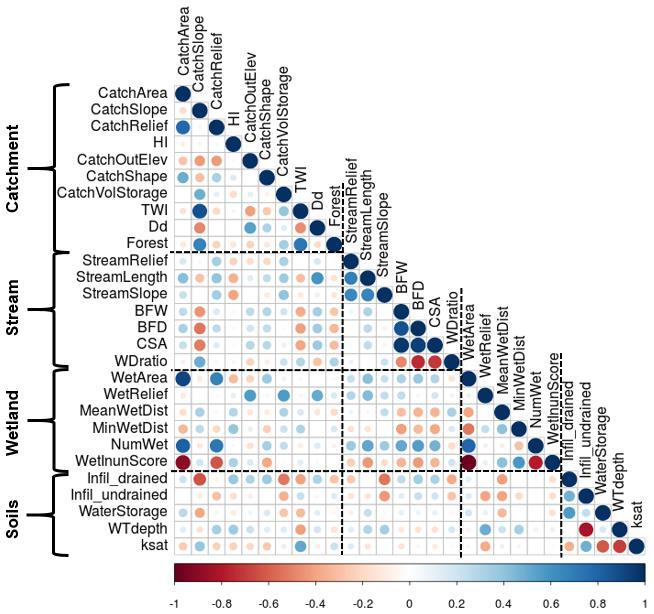
Figure F1.** Correlation matrix of landscape predictor metrics, where circle color and size represent the strength and direction (positive or negative) of the correlation between each predictor metric pair. See Table 1 for explanation of landscape predictor metrics.

REFERENCES

Daly, C., Halbleib, M., Smith, J. I., Gibson, W. P., Doggett, M. K., Taylor, G. H., … Pasteris, P. P. (2008). Physiographically sensitive mapping of climatological temperature and precipitation across the conterminous United States. *International Journal of Climatology*, *28*(15), 2031–2064. http://doi.org/10.1002/joc.1688

Lang, M., McDonough, O., McCarty, G., Oesterling, R., & Wilen, B. (2012). Enhanced detection of wetland-stream connectivity using lidar. *Wetlands*, *32*(3), 461–473. http://doi.org/10.1007/s13157-012-0279-7
